# Comparison of clinical and radiographic signs of hip osteoarthritis in contralateral hip joints of fifty working dogs

**DOI:** 10.1371/journal.pone.0248767

**Published:** 2021-03-18

**Authors:** J. C. Alves, Ana Santos, Patrícia Jorge, Catarina Lavrador, L. Miguel Carreira

**Affiliations:** 1 Divisão de Medicina Veterinária, Guarda Nacional Republicana (GNR), Lisbon, Portugal; 2 MED–Mediterranean Institute for Agriculture, Environment and Development, Instituto de Investigação e Formação Avançada, Universidade de Évora, Évora, Portugal; 3 Faculty of Veterinary Medicine, University of Lisbon (FMV/ULisboa), Lisbon, Portugal; 4 Interdisciplinary Centre for Research in Animal Health (CIISA), University of Lisbon, (FMV/ULisboa), Lisbon, Portugal; 5 Anjos of Assis Veterinary Medicine Centre (CMVAA), Barreiro, Portugal; Universita degli Studi di Padova, ITALY

## Abstract

**Objective:**

This study aimed to compare the symmetry of clinical and radiographic signs of right and left pelvic limbs of dogs with bilateral hip osteoarthritis (OA) and evaluate the association of physical findings and radiographic abnormalities.

**Patients and methods:**

One hundred pelvic limbs of police working dogs with bilateral hip OA were evaluated, following a screening program. Weight distribution, joint range of motion at flexion and extension, thigh girth, and radiographic signs were recorded and compared with the results of the contralateral limb and by breed, age, and sex with the Paired Samples T-Test and Pearson correlation coefficient, with p<0.05.

**Results:**

The sample mean age was 6.5±2.2 years, and the bodyweight of 26.7±5.3kg. No significant differences were observed when comparing weight distribution, joint range of motion, and thigh girth of left and right limbs. Weight distribution and age showed a statistically significant correlation with joint extension. The right limbs showed a significantly higher frequency of circumferential femoral head osteophyte (CFHO) regarding radiographic signs. Limbs with CFHO or caudolateral curvilinear osteophyte had significantly larger joint flexion angle (p = 0.02) and smaller extension angle (p<0.01), respectively, compared to those that did not. Age showed a significant correlation with the presence of several radiographic findings, as did different breeds.

**Conclusion:**

Clinical and radiographic signs occur symmetrically in naturally occurring hip OA in police working dogs. Several correlations were observed between the evaluations performed and differences between breeds, which can be useful in assessing and early diagnosis of hip OA.

## Introduction

Osteoarthritis (OA) poses significant welfare challenges and concerns, as it affects the quality of life, performance and implies a considerable cost in terms of healthcare [1, 2]. It is the most prevalent musculoskeletal disease in the dog and is estimated to affect around 200 000 dogs annually in the United Kingdom [3]. At least 80% of lameness cases and joint disease in companion animals are classified as OA, with 20% of middle-aged and 90% of older dogs having OA in one or more joints [4–7]. Risk factors include breed, neutered, higher bodyweight, and older than eight years [3]. Sporting and working animals are at increased risk, being exposed to repetitive loading and chronic fatigue injuries, leading to tissue damage, wear, tear, and ultimate tissue failure, resulting in clinical signs [8]. Chronic fatigue injuries are a critical predisposing condition for hip OA development, a disease commonly diagnosed in dogs, with various degrees of severity [9, 10].

Imaging plays a key role alongside the clinical review of patients with joint disease and can be done repeatedly and safely within recognized limits, which is important for the follow up of chronic conditions [11, 12]. The most common radiographic view for evaluating the hip is the ventrodorsal (VD) hip extended view, for which sedation is required for most dogs [13–15]. Main radiographic changes include femoral periarticular osteophyte formation, subchondral sclerosis of the craniodorsal acetabulum, osteophytes on the cranial/caudal acetabular margin, remodeling of the cranial and caudal acetabulum, flattening of the femoral head, and irregular widening of the femoral neck [16, 17]. The features that have been deemed of significant importance are the circumferential femoral head osteophyte (CFHO), caudolateral curvilinear osteophyte (CCO), and subchondral bone sclerosis, early radiographic signs that predict the development of the clinical signs of hip OA [18–21]. The ventrodorsal flexed view (also called frog-legged view, FL) enhances the visibility of the cranial and caudal aspects of the femoral head and neck, helping in the assessment of CFHO and CCO [21].

Weight distribution and off-loading, or limb favoring at the stance, is a commonly used subjective assessment during the orthopedic examination, but the subtle changes in posture or weight-bearing may occur in the early stage of the disease process can be easily missed with visual assessment only [22–25]. Stance analysis and weight-bearing distribution have been reported as sensitive for detecting lameness in dogs, with better results in large breed dogs [26]. Muscular atrophy is a consistent finding in OA patients and may be evident within a few weeks [10, 14, 16, 27]. The evaluation of the joint range of motion (ROM) can also be performed, including flexion and extension [10]. The evaluation of asymmetry, assessment of muscle atrophy, measurement of static weight-bearing, and ROM measurement have been described as the most valid and sensitive physiotherapeutic evaluation methods [28, 29].

This study aimed to compare the symmetry of clinical and radiographic signs of right and left pelvic limbs of police working dogs with bilateral hip osteoarthritis, and evaluate the association of physical findings and radiographic abnormalities, at the time of diagnosis. We hypothesize that multiple asymmetries are present in several of the evaluation parameters.

## Materials and methods

The study protocol was approved by the ethical review committee of the University of Évora (Órgão Responsável pelo Bem-estar dos Animais da Universidade de Évora, approval n° GD/32055/2018/P1, September 25th, 2018) and comply with ARRIVE guidelines. Written, informed consent was obtained from the Institution responsible for the animals.

This study’s sample constituted a convenience sample, similar in size to previously published reports on this topic [30–32]. The sample comprised 100 hips of 50 police working dogs with bilateral hip OA, from the population of police working dogs of the Guarda Nacional Republicana (Portuguese Gendarmerie Canine Unit), scheduled to undergo treatment of hip OA. Patients were active police working dogs, selected after screening of the Portuguese Gendarmerie Canine Unit, based on history (difficulty rising, jumping and maintaining obedience positions, stiffness and decreased overall performance), physical (pain during joint mobilization, stiffness, and reduced range of motion), orthopedic, neurological and radiographic (OFA hip scores of mild, moderate or severe) examinations compatible with bilateral hip OA.

All patients underwent medical evaluation before acquisition from multiple breeders and trainers, and starting active training to become police working dogs. Additional inclusion criteria comprised a bodyweight ≥15kg, age over 1 year should not have received any medication nor nutritional supplements for six weeks or more. Animals that did not tolerate the data collection procedures, which had any other suspected or diagnosed neurological/musculoskeletal disorder, had a diagnosis of suspected concomitant disease (ruled out through physical examination, complete blood count, and serum chemistry profile), were excluded. Subsequent treatment was randomly determined, as the animals took part in a study evaluating intra-articular therapies for OA. Two-hundred and eighty-one dogs were screened, and 231 were excluded. Sixty-three due to suspected or documented orthopedic, neurological, or concomitant disease, 50 due to having a bodyweight <20kg, 25 for having received medication in <6 weeks, and 13 for not being tolerant of data collection, and 80 due to an inability to maintain medical follow-up throughout the study, for work-related reasons.

**Fig 1:**
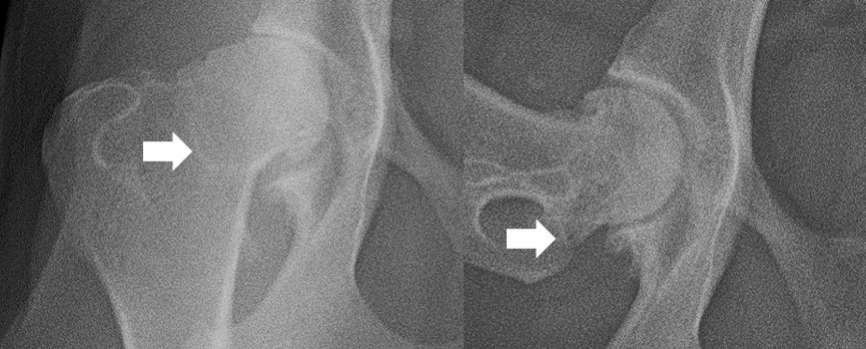
An example of a hip with caudolateral curvilinear osteophyte (arrow) on a ventrodorsal (left) and frog leg right (views).

**Fig 2:**
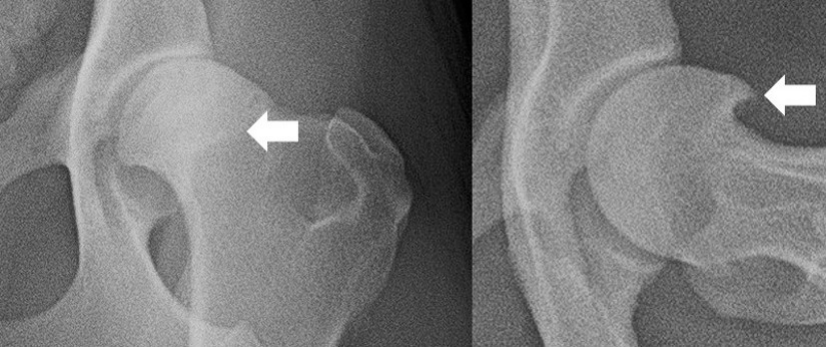
An example of a hip with circumferential femoral head osteophyte (arrow) on a ventrodorsal (left) and frog leg (right) views.

Radiographic studies were conducted under light sedation, using a combination of medetomidine (0.01mg/kg) and buthorphanol (0.1mg/kg), given intravenously. A VD extended legs view and an FL view were obtained. In the VD view, the presence of the following radiographic hip OA findings was recorded [21]: irregular wear on the femoral head, making it misshapen and with a loss of its rounded appearance; flattened or shallow acetabulum, with irregular outline; CCO; new bone formation on the acetabulum and on femoral head and neck; acetabular rim wear; subchondral bone sclerosis along the cranial acetabular edge; CFHO. In the FL view, the presence of CCO and CFHO was recorded. An example of CCO and CFHO, in VD and FL views, are presented in Figs 1 and 2, respectively.

Stance analysis was conducted with a weight distribution platform (Companion Stance Analyzer; LiteCure LLC, Newark, Delaware, United States). The equipment was placed in the center of an observation room, at least 1-meter feet from the walls. Complying with the manufacturer’s guidelines, the platform was calibrated at the beginning of each testing day and zeroed before each data collection. After an acclimatization period, animals were then encouraged to stand on to the weight distribution platform. To secure a correct position, the patient’s trainer helped ensure it placed one foot on each quadrant of the platform while maintaining a natural stance with its center of gravity and stability (measured by the platform) near the platform’s middle. When required, gentle restraint was used to maintain the patient’s head in a natural, forward-facing position. Normal pelvic limb evaluation is considered 20% of the total weight [33]. A Gulick II measuring tape was used to determine thigh girth. Measurements were made at a distance of 70% thigh length, measured from the greater trochanter’s tip, with the leg in an extended position. Animals were placed in lateral recumbency in a relaxed position [34]. ROM of the hip joints was obtained with a goniometer at extension and flexion with a flexed stifle [35].

### Statistical analysis

Normality was assessed with a Shapiro-Wilk test, and each measured parameter was compared with the contralateral limb with a Paired Samples T-Test. Measured parameters by breed and sex were compared with an Independent Samples T-Test. Evaluation of the results of radiographic imaging, digital thermography, and physical examinations were conducted without knowledge of the results of the remaining evaluations. Correlation between parameters was assessed with Pearson correlation coefficient. All results were analyzed with IBM SPSS Statistics version 20, and a significance level of p<0.05 was set.

## Results

A sample of 50 police working dogs, of both genders (30 males and 20 females), with a mean age of 6.5±2.2 years and bodyweight of 26.7±5.3kg, were analyzed. Four breeds were represented: German Shepherd Dogs (GSD, n = 17), Belgian Malinois Shepherd Dogs (BM, n = 15), Labrador Retriever (LR, n = 10), and Dutch Shepherd Dog (DSD, n = 8). They were used for four different purposes: Use of force (n = 20), drug detection (n = 14), search and rescue (n = 7), explosives detection (n = 5), and tactical intervention (n = 4). Considering OFA grading of hip joints, 35 animals were classified as mild (70%), 10 as moderate (20%), and 5 as severe (10%).

Measured age and weight values, individual limb weight distribution, thigh girth, and joint range of motion are presented in Table 1. No significant differences were observed when comparing overall measurements of left and right limbs. Significant differences were observed in weight and thigh girth in both right and left pelvic limbs (p<0.01) when comparing males to females, with male dogs having higher values. Comparing breeds, LR were significantly older than other animals (p<0.01), and GSD were significantly heavier than BM (p<0.01) and LR (p<0.01). GSD also had a significantly higher left thigh girth than BM (p<0.01), LR (p<0.01) and DSD (p = 0.04), and right thigh girth than BM (p<0.04) and LR (p<0.01). DSD had higher left joint flexion than BM and GSD (p = 0.04), while LR had a larger joint flexion angle (p = 0.05). Sex showed a correlation with weight (r = 0.5, p<0.01). Breed showed a moderate correlation with thigh girth (r = 0.4, p<0.01 for the left pelvic limb and r = 0.3, p = 0.04 for the right pelvic limb), as did sex (r = 0.5, p<0.01 for both limbs) and high correlation with weight (r = 0.8, p<0.01 for both limbs). A correlation was observed between joint extension and age (r = -0.4, p<0.04 for the left pelvic limb and r = 0.3, p<0.02 for the right pelvic limb) and weight distribution (r = 0.5, p<0.01 for the left pelvic limb).

**Table 1:** Mean scores (±standard deviation) of overall weight and age, individual stance analysis, thigh girth and range of motion (extension and flexion) measurements, and by breed and sex, of left and right pelvic limbs.

	Weight	Age	Stance Analysis		Thigh Girth		Joint Extension		Joint Flexion	
	(kg, mean±SD)	(yrs, mean±SD)	(%, mean±SD)		(cm, mean±SD)		(°, mean±SD)		(°, mean±SD)	
			Left	Right	Left	Right	Left	Right	Left	Right
Overall	26.7±5.3	6.5±2.2	19.2±4.8	18.7±4.2	30.6±2.9	30.4±2.6	149.2±9.5	150.6±7.1	55.6±4.1	56.2±4.6
German Shepherd Dog	29.9±6.4	5.7±1.8	20.0±3.9	18.4±3.6	32.5±3.5	31.8±2.7	151.1±8.1	151.6±5.9	55.2±3.5	57.1±3.5
Belgian Malinois Shepherd Dog	27.5±4.1	5.3±1.4	17.8±5.5	19.8±5.9	29.9±2.7	29.9±2.2	146.7±7.1	150.6±4.9	54.3±4.6	56.1±6.1
Labrador Retriever	24.3±2.5	8.7±2.5	19.9±5.5	18.6±3.2	28.5±2.5	28.5±2.3	147.0±14.4	148.5±10.8	55.8±3.8	54.3±3.7
Dutch Shepherd Dog	27.5±4.1	5.3±1.4	19.0±4.4	17.4±2.9	30.2±2.1	30.7±2.1	152.5±8.8	151.5±7.9	58.5±3.7	56.5±4.2
Male	29.3±5.4	6.2±2.4	19.0±5.5	19.3±4.8	31.7±2.9	31.2±2.5	149.2±7.6	151.1±4.9	55.2±3.8	57.0±4.7
Female	23.5±2.8	6.9±2.5	19.4±3.5	17.8±3.1	28.8±2.0	28.9±2.2	149.2±12.1	150.0±9.6	56.2±4.5	54.9±4.2

Regarding radiographic findings, absolute frequencies and percentages in the VD and FL views of the left and right pelvic limbs are presented in Table 2. Breed variations in Orthopedic Foundation for Animals hip scores are shown in Table 3. Comparing contralateral limbs, only the presence of CFHO, observed in the VD view, was significantly different (p = 0.03). DSD had significant differences in the frequency of CFHO in the VD view compared to GSD (p = 0.03) and BM (p = 0.04) (left and right pelvic limbs, respectively). On the FL view, differences were observed in the frequency of CFHO of BM and GSD (p = 0.02) and DSD (p = 0.04), both on the left pelvic limb.

**Table 2:** Absolute frequencies and percentages of radiographic findings in the ventrodorsal and frog-leg views of the left and right pelvic limbs.

Radiographic finding	Left				Right			
	Present		Absent		Present		Absent	
Irregular wear on the femoral head, making it misshapen and with a loss of its rounded appearance	48	96%	2	4%	47	94%	3	6%
Flattened or shallow acetabulum, with irregular outline	32	64%	18	36%	28	56%	21	42%
Caudolateral curvilinear osteophyte (CCO)	17	34%	33	66%	18	26%	32	64%
New bone formation on the acetabulum and on femoral head and neck	41	82%	9	18%	45	90%	5	10%
The angle formed at the cranial effective acetabular rim is worn away	36	72%	14	28%	41	82%	9	18%
Subchondral bone sclerosis along the cranial acetabular edge	49	98%	1	2%	49	98%	1	2%
Circumferential femoral head osteophyte (CFHO)	18	36%	32	64%	40	80%	10	20%
CCO on the Frog Leg view	18	36%	32	64%	15	30%	35	70%
CFHO on the Frog Leg view	45	90%	5	10%	43	86%	7	14%

**Table 3:** Breed variations in Orthopedic Foundation for Animals hip scores.

	OFA hip grade					
	Mild		Moderate		Severe	
Overall	35	70%	10	20%	5	10%
German Shepherd Dog	9	53%	5	29%	3	18%
Belgian Malinois Shepherd Dog	13	87%	2	13%	0	0%
Labrador Retriever	7	88%	1	13%	0	0%
Dutch Shepherd Dog	6	60%	2	20%	2	20%

A difference in the frequency of the presence of a worn cranial effective acetabular rim angle was observed in the right pelvic limb of GSD and LR (p = 0.04). Breed showed a moderate correlation with the presence of CFHO in the VD view (r = 0.302, p<0.05 for the left pelvic limb). A moderate correlation was observed between age and the presence of CCO on the FL view (r = -0.408, p<0.01 for both pelvic limbs), an irregular, misshapen femoral head (r = 0.302, p = 0.02 for the left pelvic limb), and new bone formation on the acetabulum and femoral head and neck (r = 0.312, p = 0.03 for the right pelvic limb). The joint extension was high or moderate correlated with the presence of CCO in an FL view (r = -0.506, p = 0.01 for the left pelvic limb and r = -0.439, p<0.01 for the right pelvic limb) and on the VD view(r = -0.315, p<0.04 for the right pelvic limb). Joint flexion was moderate correlated with the presence of CFHO in the VD view on both limbs (r = -0.312, p = 0.02 for the left pelvic limb and r = -0.304, p<0.04 for the right pelvic limb).

Considering animals that did or did not present CCO or CFHO on the left pelvic limb, significant differences were observed in joint extension when CCO was present in both the VD and FL views (p<0.01 for both) and joint flexion when CFHO was present in a VD view (p = 0.02). On the right pelvic limb, a significant difference was observed in joint extension when CCO was present, in both the VD and FL views, compared to when it was absent (p<0.01).

## Discussion

Hip OA is common in large breeds such as German Shepherd Dogs and Labrador, amongst others [36]. This study describes and compares several clinical and radiographic findings of different individuals in each individual and between breeds with hip OA. It also describes as, in these animals, disease derived changes occur in similar degree in both limbs since no significant differences were observed when comparing contralateral limbs. The reason for this finding is unclear and may be associated with the natural progression of the disease or with a relatively early diagnosis, the effect of a possible unilateral, or even intermittent overloading is still not noticeable.

Most hip OA signs are observed in the older population, usually in animals over 8 years, with the disease now at a chronic stage [3]. The mean age of animals included in this study was lower (6.5) years, except for LR. This result is not in line with published studies and may be associated with the fact that these animals are active working dogs, where complaints due to musculoskeletal disease are noticed early on, from its toll on gait and performance [37]. The difference in age of LR is less apparent and may be due to breed conformation, leading to a better disease tolerance, as hip OA seems to be better tolerated by animals than OA in different joints [10]. Additional reasons may be related to a less physically demanding mission of these dogs (most were product detection dogs) than the remaining animals included in the sample (mostly in search and rescue and Use of force activities).

Some of the other registered differences could be expected. GSD were significantly heavier than other breeds (BM and LR) and with higher thigh girth. Male dogs also heavier than females and with higher thigh girth. This may account for a positive correlation between thigh girth, weight, sex, and breed. The effect of weight and growth rate has been studied concerning hip dysplasia from an early stage, with heavier dogs showing a higher incidence of OA at maturity and an earlier age [38, 39]. This can partially account for the higher number of males included in this sample since they tend to be heavier and, therefore, possibly more prone to develop OA. Measuring thigh girth is also be a useful measurement, not only in the initial assessment but also as an outcome measure [34]. We have presented thigh girth measurements in dogs with hip OA, but further studies should include a control group with disease-free dogs to compare both groups’ values. Coxofemoral ROM may also be diminished, particularly during extension, although this is not a universal finding [14, 40]. Normal ROM of the hip joint in military working German Shepherd Dogs are described as 44°±6 at flexion and 155°±6 at extension [41]. In Labrador Retrievers, normal ROM been described as 50°±2 at flexion and 162°±3 at extension in one report, and 49° at flexion and 159° at extension in another [35, 42]. We have measured lower values in GSD and LR, which could be expected due to OA. Exercise has been described as having a positive effect on the severity of lameness in LR with hip dysplasia, which increased with longer exercise duration [43]. Since the animals included in the sample are all active working dogs, this inverse relationship can be present. The joint extension was one of the measurements made with higher dispersion, which may confirm the fact that ROM changes are not a consistent finding. It showed a correlation with age, which may be attributed to disease progression, as joint extension appears to be limited by the joint capsule fibrosis. This reduction in joint extension in older dogs has been described before [43]. On the other hand, hip joint flexion seems to be related to muscle mass and could increase in dogs with OA due to a loss in muscle masses [44]. As the patients in our sample did not exhibit a high degree of muscle atrophy, this increase was not observed.

Normal weight distribution for the stance analyzer is the same as for a pressure-sensitive walkway—30/30/20/20 (left thoracic limb/right thoracic limb/ left pelvic limb/right pelvic limb) [45, 46]. It has been proposed that bodyweight distribution at a stance may be an equivalent or superior measurement of pain associated with hip OA than both VI and PVF [33, 47], and dogs presenting with the disease often have slightly abducted pelvic limbs, easily noticeable when the animal is standing, increasing acetabular coverage [10]. Overall weight distribution values per limb recorded in this study were lower than the described 20% expected level. When analyzing weight distribution in individual breeds, the exception was registered in the left pelvic limb of GSD, but this may be a compensation mechanism. No significant variations were observed between breeds. This measurement’s value, particularly in response to treatment, should be the subject of further research since it was the measured parameter that recorded the least breed associated variations.

It is well established that radiographic signs’ development occurs later than the structural changes associated with OA, and symptoms must be severe before being observed on the X-ray [14, 48]. CFHO is a marked radiopaque line encircling the junction between the femoral neck and the epiphysis, along with the insertion of the joint capsule [49]. When comparing radiographic findings between contralateral limbs, only the frequency of CFHO on the VD view was significantly different. Since this is one of the radiographic predictors of future OA development [21], and these animals were only now being diagnosed, future studies will have to address whether further asymmetries between limbs develop if it was only an incidental finding. Since CFHO is usually better observed on an FL view, and the same asymmetry was not observed on the FL, this might be the case. CFHO also correlated with joint flexion, which can be explained by the changes that occur in the joint capsule during OA progression, making it less flexible. GSD also had significantly higher frequencies of this finding than BM and DSD. Interestingly, no correlation was found between weight and any of the radiographic findings considered, as could be expected, and they may rather be linked to breed variations.

CCO arises at the femoral neck’s caudodorsal part due to traction on the hip joint capsule, and it manifests as a radiopaque line. It correlates with hip subluxation and, therefore, represents a risk factor for OA development at a later stage in life [15]. A correlation between CCO and age of an irregular femoral head and new bone formation was observed and can further attest to the relationship between CCO and OA signs’ development. It also correlated with joint extension and, as seen with CFHO, may be due to the changes that occur in the joint capsule. This possibility is also reflected when considering the animals that presented CFHO or CCO and comparing them with those that did not, with significant differences in ROM evaluation, flexion, and extension.

This study presents some limitations. Ideally, a control group with non-lame dogs should be included. The interest of each of the findings in the prognosis or treatment monitoring of OA could not be determined since data was collected in a single evaluation moment. Future studies are required to evaluate these points. Additionally, the sample included a majority of dogs with mild OA. For that reason, further studies should include a larger number of dogs with moderate and severe OA to determine if similar results are obtained.

## Conclusions

The present study showed that clinical and radiographic signs occur symmetrically in naturally occurring hip OA in police working dogs. It also describes the correlation between the evaluations performed, which can be useful in evaluating and early diagnosing hip OA, as differences between the most commonly used working dogs breeds.
