# Expression of Concern: Comparison of clinical and radiographic signs of hip osteoarthritis in contralateral hip joints of fifty working dogs

**DOI:** 10.1371/journal.pone.0276122

**Published:** 2022-10-07

**Authors:** The PLOS ONE Editors

After publication of this article [1], concerns were raised about possible overlap or re-use of datasets and potential data discrepancies across a group of closely related publications [1–6]. The studies reported previously in [2–4] and [6] were not cited and discussed in [1]. In following up on these issues, the following was noted with regard to the datasets reported in these studies:

- The three articles [1–3] each report a sample of 30 male and 20 female working dogs with the same overall aggregate weight and age data. The weight and age data for the 50-animal sample in Table 1 of article [1] are the same as in Table 1 of article [2] but there are minor differences in weight and age data reported by breed and sex in Table 1 of article [1] compared to Table 1 of article [3].
- Table 1 in article [1] and Table 1 in article [3] also report individual stance analysis, thigh girth and range of motion (extension and flexion) measurements by breed and sex; in article [1] the left and right pelvic limb data are presented separately, in article [3] the left and right pelvic limb data are presented together.
- Table 2 of article [1] and Table 2 of article [3] report broadly similar findings in radiographic evaluations; in article [1] the radiographic findings are presented by left and right pelvic limbs, whereas in article [3] the data are not separated by limb.

The corresponding author stated that these articles are part of a larger project which was run with a specific population of police working dogs, a relatively homogenous sample in terms of breeds, size, and conformation, and that article [3] is a descriptive study, where clinical and diagnostic imaging findings of this group of animals with bilateral hip osteoarthritis have different characteristics to companion dogs, while article [1] follows an experimental and more academic approach which looked at each joint individually to determine if there is a level of symmetry in animals with bilateral hip OA.

The corresponding author has also indicated that because of legal restrictions, the authors are unable to provide the *PLOS ONE* Editors with the underlying individual level data for this study. The published route for requesting access to the underlying data does not seem to be functional at the time of publication of this notice.

Where differences are observed between the datasets, it has not been possible to confirm the accuracy of the data reported in this *PLOS ONE* article [1].

In light of the unresolved questions about the datasets, the *PLOS ONE* Editors issue this Expression of Concern.
